# The challenges of the low birth rate in China

**DOI:** 10.1002/puh2.8

**Published:** 2022-06-15

**Authors:** Don Eliseo Lucero‐Prisno, M. B. N. Kouwenhoven, Paolo Miguel Manalang Vicerra, Zheng Feei Ma, María José González Méndez, Angelica Joyce Abordo Gacutno‐Evardone, Emery Manirambona, Dawa Gyeltshen, Shuaibu Saidu Musa

**Affiliations:** ^1^ Department of Global Health and Development London School of Hygiene and Tropical Medicine London UK; ^2^ Faculty of Management and Development Studies University of the Philippines Open University Los Baños Laguna Philippines; ^3^ Faculty of Public Health Mahidol University Bangkok Thailand; ^4^ Department of Physics Xi'an Jiaotong‐Liverpool University Suzhou PR China; ^5^ Asian Demographic Research Institute Shanghai University Shanghai China; ^6^ Department of Health and Environmental Sciences Xi'an Jiaotong‐Liverpool University Suzhou PR China; ^7^ Department of Public Health Dalian Medical University Dalian PR China; ^8^ Department of Pediatrics Eastern Visayas Medical Center Tacloban City Philippines; ^9^ Global Health Focus Kigali Rwanda; ^10^ Jigme Dorji Wangchuk National Referral Hospital Thimphu Bhutan; ^11^ Department of Nursing Science Ahmadu Bello University Zaria Nigeria; ^12^ Global Health Focus Abuja Nigeria

**Keywords:** low birth rate, population, China, aging, population growth, economy

## Abstract

**Introduction:**

The population growth rate of China has been steadily declining owing to the low birth rate of the country. The Chinese census also indicates that the population is also rapidly ageing because of increasing life expectancy in conjunction with the low birth rate.

**Method:**

We performed a commentary to point out the challenges of the low birth rate in China. A comprehensive data search was performed in data bases such as Google Scholar and PubMed using predetermined search term.

**Results:**

The decline in birth rate is due to societal changes including increasing standard of living. Although the Chinese government has recently allowed couples to have up to three children in a major policy shift, many couples remain unwilling to have more than one child due to the high cost. An increasing fraction of Chinese women, particularly among the highly‐educated urban dwellers, no longer regard marriage and parenthood as essential aspects of life.

**Conclusion:**

If not addressed urgently, the declining young population will impact China's future socio‐economic situation, as there will be a smaller workforce relative to an increasing dependent older population. The repercussions may cascade to other spheres of China's status, including socioeconomic, security and global influence, if not addressed decisively. It is imperative that the country develops policies, strategies, and approaches to tackle this issue, including human capital development and the use of technology and innovations.

## INTRODUCTION

With its over 1.4 billion citizens, China is the most populous country in the world. China has been experiencing a declining growth rate in its population. This decline is attributed to a sustained low birth rate. The birth rate is defined as the annual number of live births per 1000 individuals in a given population. In China, there were 7.52 births per 1,000 people in 2021, a further decline from the rate of 8.52 per 1,000 persons of the previous year [1]. The said birth rate in 2021 was the lowest since 1949. China's birth rate has been falling since the beginning of the 21^st^ century, with authorities recently claiming a fertility rate of merely 1.8, while the actual figure may likely to be closer to 1.1 [2]. The imposition of the birth control policies further contributed to the decline. The government implemented the one‐child policy in 1979 to control the seemingly booming population, even if there was already an observed decline prior to this.

The economic miracle that China has shown the world — an unprecedented economic growth in a time span of just a few decades, has provided the impetus for the improvements of the lives of its population. The double‐digit economic growth rate fueled the rapid developments in infrastructure within the country. Aside from national developments, both personal and family wealth also grew exponentially. A substantial fraction of the population now lives in decent warm or air‐conditioned houses, has better access to health, has ample food, has access to good health services, clean water, good public utilities, and to education. A large proportion of the older population was raised from below the poverty line. The economic developments of the recent decades resulted in better health of the population, and have consequently resulted in an increasing life expectancy and a lower rate of mortality from various illnesses. The death rate also slowed down, resulting in a higher proportion of older people growing toward much advanced ages.

These population dynamics contribute toward an ageing population. This context has been noted by policymakers to be impactful to the economy because the increasing dependency ratio of older adults is inversely proportional to the working age population of the society. A country which relies on its extensive workforce as the engine of economic development would suddenly be slowing down. Wary of the immediate future, this scenario led the country to change the policy from allowing one child to allowing two children per couple in the year 2016, in an attempt to reverse the trend in the birth rate. After China's recent census data showed a steep decline in birth rates [1, 3], the purpose of this shift in policy seemingly did not serve its purpose, thereby forcing the country to quickly shift its policy again, to allow couples to have up to three children.

### Impact of low birth rate in China

The 2021 statistics shows that around 11 million babies were born [1]. This showed a significant decrease from the 18 million in 2016, which hitherto has been considered the lowest number of births recorded since the 1960s at the time [3]. This poses a serious challenge to the future development of China. In recent decades, China's economic growth and prosperity have been supported by its large working‐age population [4]. Its workforce provided the manufacturing base that produced and supplied many of the products consumed by the world. Fueled by a neoliberal global order, this resulted in double‐digit economic growth rate of the country. The income generated, transformed the country almost overnight, which allowed China to make substantial infrastructural investments, including the construction of the world's largest network of bullet trains. Starting from the production of simple products such as plastic household items, technological innovations eventually resulted in the production of high‐end electronics and electric cars. This further increased the revenue of the country, as it leveraged on cheap labor and an increasingly educated and technologically‐savvy workforce.

The ongoing demographic shift poses a number of challenges to China's future social and economic development. Labor and skills shortage is expected as the number of working‐age adults dwindle each year that may result to a slowing of the economy [5]. This will also be aggravated by the increasing standard of living vis‐à‐vis the cost of Chinese labor, as many workers become highly educated. Factory owners are faced with the high salaries of their skilled workers, and some have decided to move their business to cheaper locations outside China, such as in neighboring ASEAN countries.

A dwindling economy may also have global significance, and may affect foreign policy influence [6]. With a reduced economic capacity, its flagship program of Belt and Road Initiative may diminish some of its influencing capacity. There may also be geopolitical implications if neighboring India quickly surpasses China as the world's most populous country in the future [6].

The impact of the changing demographic structure will also affect social structures, since a smaller productive workforce has to care for a larger number of dependents from the older population. There may be a need to institutionalize the care of the older adults, thus eroding the traditional family‐centered culture that is quite strong in most Asian societies. Given this financial pressure, the disinterest of young adults with having a child will become more palpable. Marriage will become less enticing, as already experienced in many European countries. These all have the potential to change the family landscape of China in the near future.

Even if China were to prevent the adverse economic effects of a declining workforce, social stagnation would remain a possibility as the population becomes more risk‐averse [2]. Without a young population, the country may lose some of the strengths of its social dynamics, which is a major factor in national development. In a society where the share of older adults Eclipse the younger aged workforce, as observed in Japan in 2012, the population tends to seek for more safety and security [2]. The population becomes averse to risks rather than manages it and work toward innovation. The projections of China's future demographic makeup using population modeling show that the country will undergo a similar inversion around the year 2034. There is a possibility that this will occur even sooner, because China's fertility rate is further decelerating [2].

### Factors associated with low birth rate in China

The government has been attempting to prevent the looming birth rate havoc by allowing couples to have multiple children. Notably, the adult population of the developed urbanized regions is inclined toward fewer marriages and fewer children, as shown by China's most recent statistical yearbook, illustrating the gravity of the situation [7]. Even though the government encourages couples to have more children, evidence shows that one out of two parents prefers to have only one child [7]. As a major social institution for raising children, marriage rates are a contributing factor to China's low birth rate. Marriage registration numbers fell for seven consecutive years in 2020, with only 8.1 million couples getting married — a 12% reduction from the previous year and a massive drop from the 13.4 million marriages in 2013 [7].

An increasing number of Chinese women, especially those that are highly educated, and in urbanized regions, consider it undesirable or unnecessary to become a parent, resulting in resisting the pressure from their own parents to bear children [8]. This drop may also be an indicator of delaying marriages to a latter age. Postponed marriages reduce the possibility of parenthood, because of physiological reasons, or simply due to a focus on companionship as the reason for living together. In addition, the country has also seen an increasing divorce rate, although there was a recent dip which is concomitant with the drop in the number of weddings [9].

The one‐child policy has shaped, to some degree, the way many Chinese couples think of children. Experts may disagree on the extent to which the policy affected China's long‐term fertility rate, but most agree that the economic growth in the recent decades has also hastened the country's fertility decline [10]. Those raised as a single child often also believe that they should also have a single child themselves. They tend to feel that the child deserves to have a good life through concentrating all the family resources on him or her.

### Recommendations

The impact of the low birth rate on the future of China's social and economic developments may be substantial. China is currently making efforts to avert this trend; it should rethink its population policies and social policies to address and adjust to the changing times. This includes regularly revisiting its birth control policy, and could include encouraging more marriages. There are now precedent countries which can provide important lessons on which paths to take and which mistakes not to make. Policies that provide incentives to couples in having more children may be improved. This could include raising child allowances and benefits, increasing maternity and paternity leaves, and improving housing support and child education support. Lastly, innovation is the only way forward when navigating a changing workforce and economy [11, 12]. Human capital development and the use of novel technologies such as the use of robots in manufacturing industries and innovative approaches towards economic development will be able to cushion the impact of these demographic changes by employing new paradigms in its policies.

## CONCLUSION

China's population has been its economic driver, as well as the determinant of its global stature and importance. If the trend of a shrinking young population and an increasing elderly population continues, the country may be facing many challenges that other developed countries in East Asia and the West experienced. All these changes may dampen China's international influence.

## CONFLICT OF INTEREST

The authors declare that they have no known competing financial interests or personal relationship that could have appeared to influence the work reported in this paper.

## AUTHOR CONTRIBUTION

Don Eliseo Lucero‐Prisno, M. B. N. Kouwenhoven and Shuaibu Saidu Musa conceived the idea. Don Eliseo Lucero‐Prisno, M. B. N. Kouwenhoven, Paolo Miguel Manalang Vicerra, Zheng Feei Ma, María José González Méndez, Angelica Joyce Abordo Gacutno‐Evardone, Emery Manirambona, Dawa Gyeltshen and Shuaibu Saidu Musa collected and analyzed the data and information and rotated in writing different versions of the drafts. All authors read and approved the final manuscript.
